# Gender Differences in Joint Biomechanics During Obstacle Crossing with Different Heights

**DOI:** 10.3390/bioengineering12020189

**Published:** 2025-02-16

**Authors:** Chenyan Wang, Yuan Guo, Weijin Du, Zhiqiang Li, Weiyi Chen

**Affiliations:** 1 College of Mechanical Engineering, Taiyuan University of Technology, Taiyuan 030024, China; wangccccyyy@163.com; 2 College of Artificial Intelligence, Taiyuan University of Technology, Taiyuan 030024, Chinadwj18335100390@163.com (W.D.); 3 Shanxi Key Laboratory of Material Strength & Structural Impact, Taiyuan University of Technology, Taiyuan 030024, China; lizhiqiang@tyut.edu.cn; 4 Shanxi Research Center of Basic Discipline of Mechanics, Taiyuan University of Technology, Taiyuan 030024, China; 5 National Demonstration Center for Experimental Mechanics Education, Taiyuan University of Technology, Taiyuan 030024, China

**Keywords:** gait, gender, obstacles, kinematics, kinetics

## Abstract

Identifying gender-related gait changes offers valuable insights into the role of gender in motor control. It is anticipated that more difficult gait tasks (obstacle crossing) may reveal gender-specific effects on gait parameters. The present study aimed to explore the gait adaptations of male and female participants when stepping over obstacles of 0 cm, 13 cm, 19 cm, and 26 cm in height. A total of 12 male and 12 female participants were recruited. The Vicon motion capture system and AMTI force plates were utilized to obtain the gait parameters. Moreover, spatiotemporal parameters were investigated. Two-way repeated ANOVA (gender × obstacle height) and three-way repeated ANOVA (gender × obstacle height × leg) were performed to compare gait parameters, respectively. Correlations between maximum joint angle and obstacle height were also evaluated. Significant interactions were observed for leading leg swing time, maximum hip extension angle, maximum knee flexion angle, and maximum ankle plantarflexion angle (gender × obstacle height). There were some differences in gait parameters between males and females in the unobstructed gait, and these changes became more evident as obstacle height increased. This study also identified significant differences in gait parameters between leading and trailing legs when stepping over the obstacle.

## 1. Introduction

It is generally believed that there are inherent differences between the walking patterns of males and females [1]. The gender of a walking person can be recognized by observing the movement of their joints [2]. Certain diseases occur that are gender-specific, and this may be related to differences in walking mechanics [3]. However, some studies have indicated that there are no substantial differences in gait variables between males and females [4]. Although previous studies have reported gender-specific gait results, the present study provides a further comprehensive exploration of parameters, including spatiotemporal, joint kinematics, and kinetic parameters.

Tripping is a common cause of falls in adults [5], often occurring in the presence of fixed and visible obstacles. During everyday life, stepping over obstacles of different heights (requiring the adjustment of gait parameters) occurs frequently, including walking over crossbars on the road or outdoor sports. Compared to unobstructed gait, crossing obstacles require more muscular, cognitive, and sensory demands; therefore, they may be associated with a higher risk of falls [6]. It is also necessary to integrate sensory information such as optical flow and proprioception when crossing obstacles in order to adequately plan and adjust these movements [7]. While approaching the obstacle, participants collected visual information (e.g., height and position of the obstacle) to adjust their foot position and successfully cross the obstacle [8]. If foot position was not adjusted in time, the presence of obstacles could be detrimental to stability. Stepping over the obstacle increased gait challenge, which increased systematically with an increase in obstacle height, and it was more challenging to fully describe the effect of task difficulty on gait characteristics. Adaptation to obstacle height when crossing an obstacle may reveal gait changes that are not apparent in unobstructed gait. In young adults, obstacle height produces a graded response, with higher obstacle height corresponding to longer stance, swing, and double limb support times [9]. Many studies have investigated the effects of obstacles on spatiotemporal parameters of a given population. In contrast, relatively little work has examined the interaction between gender and obstacle height. The strategies that males and females choose to adopt when crossing obstacle are still worthy of further exploration.

The purpose of this study was to assess the effect of gender and obstacle height on gait parameters. The parameters of interest were those that may differ between males and females due to structural differences. The first hypothesis was that females would exhibit different gait parameters (e.g., shorter stride length and slower speeds) compared to males in all gait conditions. The second hypothesis was that the spatiotemporal, kinematic, and kinetic parameters would alter for both genders at different obstacle heights. Moreover, it was also hypothesized that the difference between males and females would change as the height of the obstacle increased.

## 2. Materials and Methods

### 2.1. Participants

A total of 24 adults, 12 males (age 25.17 ± 2.44 years; mass 70.30 ± 7.59 kg; height 1.74 ± 0.05 m; BMI 23.09 ± 2.27 kg/m^2^) and 12 females (age 26.50 ± 1.73 years; mass 53.71 ± 4.79 kg; height 1.60 ± 0.06 m; BMI 20.88 ± 1.75 kg/m^2^), volunteered for this study. All participants were healthy and self-reported no history of joint injury, joint instability, or musculoskeletal disorders within the past six months. The protocol was approved by the University Research Ethics Committee, and informed consent was obtained from all participants prior to testing (Approval No. TYUT202105003).

### 2.2. Testing Protocol

Kinematic data were collected at 100 Hz using a 6-camera Vicon motion capture system (Vicon, Oxford, UK). The Vicon Plug-in Gait markers (16 light-reflecting markers of 14 mm diameter) were placed on the pelvis, shank, thigh, and foot to determine the kinematics of the extremity segments during gait. Participants wore tight shorts to ensure that all reflective markers were securely attached to the bony landmarks. Kinetic data were collected at 1000 Hz from two embedded force plates (AMTI, Watertown, MA, USA).

**Figure 1:**
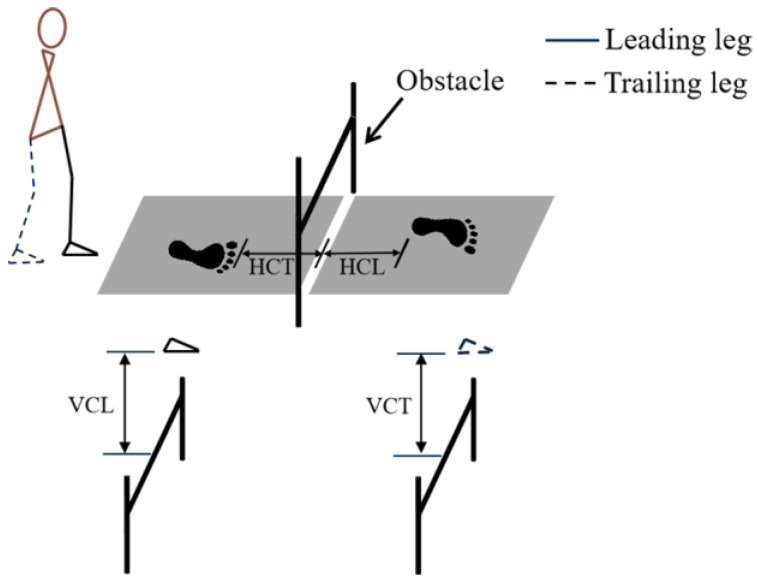
Schematic details of the obstacle. VCL: vertical distance between the obstacle and the left heel marker; VCT: vertical distance between the obstacle and the right heel marker; HCL: horizontal distance between the obstacle and the left heel marker; HCT: horizontal distance between the obstacle and the right toe marker.

Prior to formal data collection, participants were instructed to walk for several minutes to familiarize themselves with the experiments. Participants were then instructed to complete an obstacle crossing at four different heights as follows: unobstructed, 13 cm (low), 19 cm (medium), and 26 cm (high). Additionally, participants completed all gait trials barefoot at a self-selected comfortable walking speed. In the obstacle-crossing condition, the obstacle was placed in the middle of two embedded force plates, and participants stepped over the obstacle without contacting it (Figure 1). The obstacle consisted of two adjustable upright standards and a plastic crossbar (2.5 cm in diameter, 1.0 m in length).

In this experiment, the gait cycle was considered as two consecutive heel strikes of the right foot; thus, the left foot was the first foot to cross over the obstacle and was defined as the leading foot. Furthermore, the right foot’s first contact and toe-off times were determined by evaluating the data from the first force plate, and the second contact time was ascertained by the coordinate change in the heel marker. Five trials were conducted for each gait condition, and three trials were randomly selected for further study.

### 2.3. Data Analysis

The complete gait cycle was time-normalized to 100 and an average of the three trials for each obstacle condition was obtained. Similarly, the swing phase of the leading leg and the stance phase of the gait cycle were time-normalized to 100, respectively.

The spatiotemporal gait characteristics including stride length, stride time, stance time, swing time, and speed were analyzed for all four gait conditions. In addition, the vertical heel-clearance of the leading foot (VCL) and trailing foot (VCT) was determined as the vertical distance between the obstacle and the left or right heel marker, respectively, when the heel was directly above the obstacle [10,11]. The horizontal clearance of the leading foot (HCL) was considered as the horizontal distance between the obstacle and the left heel marker at the moment of heel strike. Meanwhile, the horizontal clearance of the trailing foot (HCT) was regarded as the horizontal distance between the obstacle and the right toe marker at the moment of toe-off [12].

The ground reaction force (GRF) data were filtered using a zero-lag, fourth-order, low-pass Butterworth filter with a 20 Hz cut-off frequency [13]. The GRF data were normalized to bodyweight (% BW) to allow for the comparison between subjects and genders [14]. The GRF data were analyzed using eight values. Three values were identified for the medial-lateral component, the initially occurring positive peak (Fx_PP_), and two consecutive negative peaks (Fx_N1_, Fx_N2_) of the stance phase. From the anterior–posterior curve, two peaks were determined, the initial braking force (Fy_BF_) and propulsion force at the end of stance (Fy_PF_). In addition, the vertical direction curve was characterized by two peaks, including the first peak at heel strike (Fz_FP_) and the second peak toward the end of stance (Fz_SP_) [15]. The downfall between the two peaks was also described (Fz_DF_) [16].

### 2.4. Statistical Analysis

All statistical analyses were performed using SPSS 25.0 (SPSS Inc., Chicago, IL, USA), and the significance level was set at *p* < 0.05. A two-way repeated ANOVA was adopted to explore the influence of two main factors, gender (2 levels: male, female) and gait condition (4 levels: unobstructed, low, medium, high obstacle), as well as their interactive effect on spatiotemporal parameters. Similarly, for the assessment of the maximum joint angles of the trailing leg during the complete gait cycle and the values of GRF, a two-way repeated ANOVA was also used to investigate effects of gender and gait condition. To further examine the effects of gender group, gait condition, and leg (2 levels: leading leg, trailing leg) on the maximum hip/knee/ankle flexion angle during the swing phase, a three-way repeated ANOVA was performed. The Bonferroni post hoc test was used when statistical analyses showed significant interactions and main effects. Additionally, Spearman’s rank order was applied to evaluate the relationship between the maximum joint angle of the trailing foot and obstacle height.

## 3. Results

### 3.1. Spatiotemporal Parameters

The effects of gender and obstacle height and the gender × obstacle interaction on the spatiotemporal parameters are presented in Table 1. Regarding the gender factor, the female group showed shorter stride length and lower speed than the male group. For the main effects of obstacle height, stride length, VCL, VCT, and HCL for stepping over the obstacle (low, medium, or high) were significantly higher than those of unobstructed gait. Moreover, there was a gender × obstacle interaction for the swing time of the trailing leg. The results indicated that when participants crossed obstacles (low, medium, or high), the swing time of the trailing leg was significantly increased in females compared to males (*p* < 0.05), while no effect of gender difference was shown in the unobstructed gait (*p* > 0.05).

### 3.2. Joint Angles

**Figure 2:**
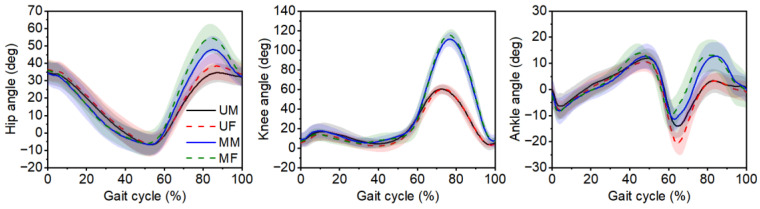
Trailing leg joint angles during the complete gait cycle for the unobstructed and medium obstacle gait. The shaded band shows standard deviation. UM: unobstructed gait for male participants; UF: unobstructed gait for female participants; MM: medium obstacle gait for male participants; MF: medium obstacle gait for female participants. Note: To increase the readability of the images, the joint angles when stepping over low and high obstacles are not shown in the figure.

The lower extremity joint angles of the trailing leg for male and female participants during the complete gait cycle for unobstructed and medium obstacle gait are shown in Figure 2. The results showed similar trends in joint angles for male and female participants. Throughout most of the gait cycle, the medium obstacle gait demonstrated greater hip flexion, knee flexion, and ankle dorsiflexion angles during the second half of the gait cycle compared to the unobstructed gait.

**Figure 3:**
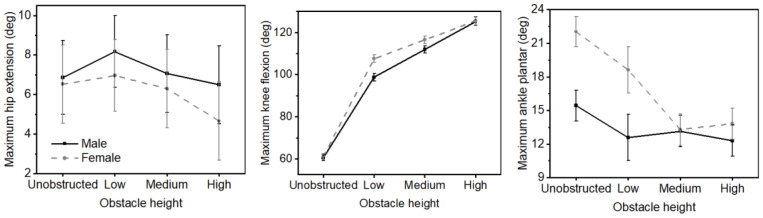
Gender × obstacle interaction for joint angle. Male participants (black solid line), female participants (gray dashed line). Error bars represent the standard error.

The statistical analyses of the peak joint angle were listed in Table 2. For the main effects of obstacle height, the maximum hip flexion angle increased with the increase in the height of the obstacle. Regarding the gender × obstacle interactions (Figure 3), female participants had a higher knee flexion angle than male participants in the low obstacle gait condition, while no differences between genders were observed in the other obstacle conditions. Furthermore, a significant interaction was also observed for the maximum ankle plantarflexion angle.

**Figure 4:**
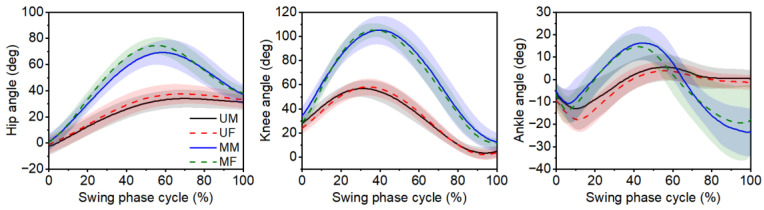
Leading leg joint angles during the swing phase for the unobstructed and medium obstacle gait. The shaded band shows standard deviation. UM: unobstructed gait for male participants; UF: unobstructed gait for female participants; MM: medium obstacle gait for male participants; MF: medium obstacle gait for female participants. Note: To increase the readability of the images, the joint angles when stepping over low and high obstacles are not shown in the figure.

The lower extremity joint angles of the leading leg for male and female participants during the swing phase for unobstructed and medium obstacle gait are shown in Figure 4. The results indicated similar trends exist in joint angles for male and female participants. The results further showed that medium obstacle gait had greater hip and knee flexion angles during the swing phase compared to the unobstructed gait. However, when stepping over the obstacle, the ankle angle patterns of male and female participants changed during the terminal swing phase, that is, the ankle joint varied from dorsiflexion to plantarflexion.

**Figure 5:**
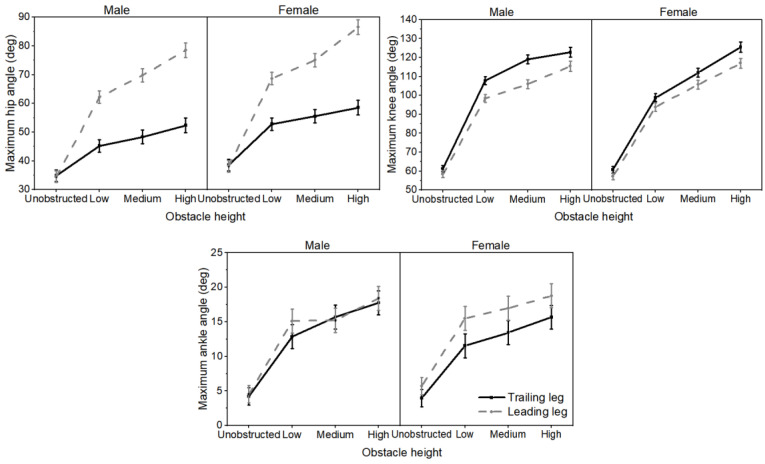
Gender × obstacle × leg interaction for joint angle. Trailing leg (black solid line), leading leg (gray dashed line). Error bars represent the standard error.

The three-way repeated ANOVA revealed significant main effects for obstacle height on the maximum hip/knee flexion angles and ankle dorsiflexion angles (Figure 5). The leg had a significant main effect on the maximum hip/knee flexion angle. The angle of leading leg was significantly lower than that of the trailing leg in terms of maximum knee flexion angle; however, there was no significant difference between the leading and trailing leg in ankle flexion angle. Additionally, the results indicated that the hip flexion angle of the leading leg was greater than that of the trailing leg during the obstacle gait (low, medium, or high). No differences in hip flexion angle between the leading and trailing leg were observed for either male and female participants in the unobstructed gait.

### 3.3. Relationship Between Joint Angle and Obstacle Height

The relationship between joint angle and obstacle height is shown in Table 3. The maximum hip flexion angle correlated with the obstacle height (r = 0.629/*p* < 0.001 for male and r = 0.632/*p* < 0.001 for female). The maximum knee flexion angle was significantly associated with obstacle height (r = 0.921/*p* < 0.001 for male and r = 0.932/*p* < 0.001 for female). Additionally, the maximum ankle dorsiflexion angle correlated with obstacle height (r = 0.436/*p* < 0.001 for male and r = 0.530/*p* < 0.001 for female).

### 3.4. Ground Reaction Force

**Figure 6:**
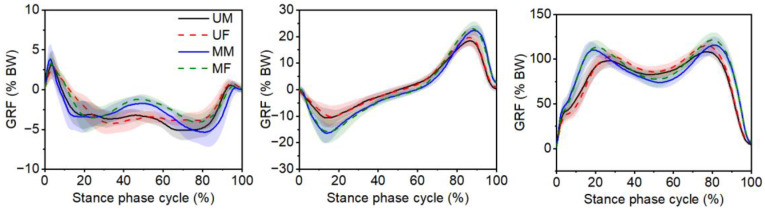
Ground reaction force (GRF) during the stance phase cycle under the unobstructed and medium obstacle gait. The shaded band shows standard deviation. UM: unobstructed gait for male participants; UF: unobstructed gait for female participants; MM: medium obstacle gait for male participants; MF: medium obstacle gait for female participants. Note: To increase the readability and simplicity of the images, the GRF when stepping over low and high obstacles is not shown in the figure.

The GRF data along the medio-lateral (Fx), anterior–posterior (Fy), and vertical (Fz) directions during the stance phase are presented in Figure 6. The results indicated that the GRF curves of male and female participants had similar trends in the medio-lateral, anterior–posterior, and vertical directions. During the 28–66% of stance phase, the Fx in the medium obstacle gait was lower than that in the unobstructed gait. The braking force was greater during the first half of the stance phase when stepping over medium obstacle compared to the unobstructed gait. In addition, the medium obstacle gait had a higher propulsion force at the terminal stance phase than the unobstructed gait. During 1–28% and 75–100% of stance phase, the Fz in the medium obstacle gait was greater than that in the unobstructed gait.

The peak GRF variables for male and female participants in different gait conditions are shown in Table 4. Regarding the gender factor, male participants exhibited greater Fx_N2_ and lower Fz_SP_ than female participants. Moreover, the main effect of obstacle height was significant for the Fx_PP_, Fx_N1_, Fy_BF_, Fy_PF_, Fz_FP_, Fz_SP_, and Fz_DF_. The interaction effects between gender and obstacle height on peak GRF variables were not statistically significant (*p* > 0.05).

## 4. Discussion

The purpose of this study was to investigate the effects of gender on the kinematics and kinetics of the lower extremity joints when stepping over unobstructed, low, medium, and high obstacles. There were more similarities than differences in gait spatiotemporal parameters, kinematics, and kinetics variables between males and females.

The stride length varied by gender, with females walking shorter strides, and similar results have been reported in overground walking studies on different genders [17,18]. The smaller stride length for females than males may be due to differences in height. There was no significant difference in stride time between male and female participants. Moreover, since gait speed is the quotient of stride length and stride time, it is not surprising that male participants exhibited significantly higher speeds than female participants [19]. The level of difficulty (i.e., obstacle heights) may affect the alertness of participants when performing the task, i.e., more caution is taken when stepping over taller obstacles [20]. During the obstacle crossing, participants adapted their gait to their physical condition by slowing down in order to perform the task cautiously. The reduced speed may be reflected in the reduced extrapolated center of mass, indicating that a conservative strategy was adopted when stepping over the obstacle, as confirmed by the absence of tripping in all gait conditions. Moreover, it has been suggested that there is a relationship between gait speed and the fear of falling [21], so the higher the obstacle, the slower the speed to avoid falling.

This result also indicated that more time was required (i.e., increased stride time) to cross the obstacle, and that an increase in the swing time of the leading leg was accompanied by an increase in the stance time of the trailing leg when stepping over the obstacle. The single leg stance phase, during which weight is redistributed, can be considered the most challenging step when the body is hovering over the obstacle [22]. It should be noted that this extended time of crossing the obstacle (i.e., single leg stance) may lead to a greater risk of falling in an unstable situation [23]. The interaction of gender and obstacle height based on the spatiotemporal parameters was also investigated. There was a significant difference in the leading leg swing time between male and female participants in the obstacle-crossing gait, but no such difference in the unobstructed gait. The results suggested that the overground gait condition was insufficient to reveal gender-related changes. However, differences were observed, not only for the high obstacle but also for the low and medium obstacle, suggesting that females may be more cautious when faced with difficult tasks. Mengarelli et al. revealed that female participants exhibit greater contraction activity and adopt a more complex muscular strategy during gait [24]. Muscle co-activation contributes to increasing limb stability during the double-to-single support transition [25]. It can be inferred that female participants require more muscle co-activation when facing more challenging obstacles. Therefore, it could be useful to develop separate movement patterns for males and females.

VCL and VCT are important parameters for detecting the success of crossing the obstacle. The progressive increase in VCL and VCT as a function of obstacle height in all male and female participants reflects their ability to recognize and adjust behaviors according to the level of task challenge. However, this strategy may be less efficient from an energetics perspective [26] and may place higher balance and stability demands on the leg [10]. Hence, the increased VCL and VCT could be an unstable behavior that may be dangerous when stepping over the obstacle, especially at higher obstacles. Controlling the trajectory of the trailing leg over an obstacle is a separate control process from that of the leading leg, and in the absence of visual use, the trailing leg is more likely to contact the obstacle and thus trip and fall [27]. In this study, the vertical heel-clearance of the trailing leg was higher than that of the leading leg, which may indicate that the trailing leg is more prone to tripping when crossing the obstacle.

Crossing the obstacle is mainly accomplished by flexing the three joints of the leading and trailing leg, even though the proximal joints, hip, and knee flex more than the ankle joint. Cho et al. reported that females showed a significantly greater hip flexion angle during walking [28]. Although not significant, the present study found similar results in that female participants had greater hip flexion angles in all gait conditions. This may be due to the structural differences that females exhibit at the hip and knee, which predispose them to having different movement patterns as well [29]. In addition, another focus of this study was to explore the interaction of gender and obstacle height on gait biomechanics. Male and female participants had similar knee flexion angles during walking, but only when stepping over low obstacles did the knee flexion angle change significantly in females compared to males. For the ankle plantarflexion angle, there was a significant difference between male and female participants during the unobstructed gait. However, as obstacle height increased, this difference gradually disappeared. This may be attributed to the fact that physiological and neuromuscular differences between males and females appear to influence the effects of different obstacle heights on lower extremity joint kinematics. Therefore, the choice of the potential small muscle training or rehabilitation means should be different in males and females.

In this study, there was a clear difference in ankle angles between the leading and trailing leg when stepping over the obstacle. Particularly in the terminal swing phase of the leading leg, the ankle joint changed from dorsiflexion to plantarflexion. The fact that there were differences in the ankle joint angle of the leading leg but not the trailing leg suggested a difference in crossing obstacle compared to the unobstructed gait. In general, these results imply that participants used different strategies to control their leading and trailing legs when stepping over the obstacle. Thus, the interaction of gender × obstacle × leg on joint angle during the swing phase was further investigated. The results showed that legs had a main effect on maximum hip/knee flexion angle. Additionally, legs and obstacle heights had an interaction effect on maximum hip flexion angle. This study indicated that the leading and trailing leg have some independence when stepping over obstacles. Furthermore, the different performance of the leading and trailing leg in crossing obstacle can be attributed to the fundamental phenomenon, which is that the leading leg relies on vision, while the trailing leg relies on memory [30,31].

The strong positive correlation between maximum knee flexion angle and obstacle height was observed in both male and female participants, so the higher the obstacle, the greater the knee flexion angle, which means more muscle forces are required. A moderate positive correlation was found between hip flexion/ankle dorsiflexion and obstacle height. The increased joint angles may be attributed to the result of adjusting joint kinematics when stepping over obstacle. It is presumed that prolonged obstacle crossing may accelerate muscle fatigue.

In this study, similar results were observed between males and females for the second peak of vertical GRF during the unobstructed gait, and in addition, females exhibited a greater second peak vertical GRF in obstacle crossing. The results of the present study also indicated that there was a significant difference between males and females in the second negative peak of medial-lateral GRF. Furthermore, as obstacle height increased, the GRF variables were significantly different from those of the unobstructed gait, except for the medial-lateral GRF variables (the first and second negative peaks). This study showed that during the first half and terminal portion of the stance phase, the braking/propulsion amplitudes in obstacle-crossing gait were greater than those in the unobstructed gait. It can be assumed that a greater effort is required to push the body’s center of mass forward when stepping over the obstacle [32]. The first peak of the vertical GRF is primarily related to body mass and acceleration, while the second peak is associated with the altered neuromuscular control of the lower extremity [33]. Thus, the increase in the first and second vertical GRF peaks during the obstacle-crossing gait may be due to changes in body acceleration and neuromuscular control after seeing the obstacle.

There are some limitations to this study that must be acknowledged. This study did not address other kinetic variables (i.e., joint moments) and muscle activities during obstacle crossing. Combining kinematic variables, kinetic variables, and muscle activities may provide further insights into the effects on the lower extremity when stepping over obstacle. Moreover, the sample size for this study was relatively small, and more participants should be recruited for further study.

## 5. Conclusions

The current study extends the knowledge based on previous findings by not only reporting the differences between males and females in stepping over obstacle, but also by elucidating the interaction between gender and obstacle height. There were significant differences for certain gait parameters (e.g., stride length and speed) between males and females. The results also showed that the variability of some gait parameters between genders varied with obstacle height. The present study provides a window into further understanding the physiological changes that occur in both genders in response to different task difficulties that may not be apparent in the unobstructed gait.

## Figures and Tables

**Table 1:** Mean and standard deviations of spatiotemporal parameters. The last three columns show the *p*-values for the main effects of the gender and obstacle height and the gender × obstacle interaction, respectively.

		Unobstructed	Low	Medium	High	Gender	Obstacle	Gender × Obstacle
Stride length (mm)	M	1269.3 (46.2)	1300.0 (53.5)	1295.4 (53.0)	1309.0 (68.4)	0.004	<0.001	0.300
	F	1195.2 (35.4)	1263.1 (51.5)	1236.0 (55.8)	1254.8 (43.3)			
Stride time (s)	M	1.15 (0.07)	1.30 (0.11)	1.35 (0.11)	1.48 (0.10)	0.104	<0.001	0.059
	F	1.15 (0.12)	1.39 (0.18)	1.46 (0.18)	1.66 (0.25)			
STT (s)	M	0.71 (0.04)	0.80 (0.06)	0.82 (0.07)	0.89 (0.07)	0.230	<0.001	0.309
	F	0.72 (0.07)	0.85 (0.11)	0.88 (0.11)	0.95 (0.19)			
SWL (s)	M	0.47 (0.03)	0.56 (0.05)	0.59 (0.05)	0.64 (0.04)	0.037	<0.001	0.018
	F	0.47 (0.04)	0.62 (0.08)	0.64 (0.08)	0.73 (0.10)			
Speed (mm/s)	M	1112.5 (74.0)	1004.6 (88.4)	965.0 (96.9)	888.4 (83.7)	0.016	<0.001	0.191
	F	1052.0 (108. 5)	922.9 (113.4)	856.7 (90.1)	770.1 (114.7)			
VCL (mm)	M	62.3 (12.1)	105.1 (47. 7)	112.1 (36.7)	129.3 (33.3)	0.988	<0.001	0.927
	F	56.4 (13.0)	104.6 (39.4)	114.7 (45.8)	132.3 (50.2)			
VCT (mm)	M	241.8 (23.5)	307.3 (47.2)	330.9 (46.6)	346.5 (59.2)	0.191	<0.001	0.770
	F	226.8 (14.1)	301.4 (38.3)	308.7 (38.9)	323.3 (41.5)			
HCL (mm)	M	166.4 (53.3)	188. 6 (46.9)	187.7 (32.7)	189.0 (36.8)	0.077	0.019	0.742
	F	176.2 (44.4)	220.4 (42.8)	216.7 (30.9)	217.5 (28.0)			
HCT (mm)	M	219.7 (40.5)	226.9 (32.7)	225.3 (32.1)	226.2 (34.1)	0.333	0.268	0.843
	F	202.8 (43.8)	216.3 (40.7)	208.8 (42.9)	218.8 (24.4)			

STT: Stance time of trialing leg; SWL: Swing time of leading leg; VCL: vertical heel-clearance of the leading foot; VCT: vertical heel-clearance of the trailing foot; HCL: horizontal clearance of the leading foot; HCT: horizontal clearance of the trailing foot; M: male; F: female.

**Table 2:** Mean and standard deviations of the peak joint angle of the trailing leg during the complete gait cycle. The last three columns show the *p*-values for the main effects of the gender and obstacle height and the gender × obstacle interaction, respectively.

		Unobstructed	Low	Medium	High	Gender	Obstacle	Gender × Obstacle
Max hip flexion	M	36.0 (5.8)	45.1 (7.2)	48.3 (7.8)	52.3 (8. 7)	0.063	<0.001	0.161
	F	39.1 (7.5)	52.8 (8.5)	55.7 (9.4)	59.0 (10.6)			
Max hip extension	M	6.9 (5.9)	8.2 (5.5)	7.1 (6.2)	6.5 (6.4)	0.701	<0.001	0.021
	F	6.5 (7.0)	7.0 (7.0)	6.3 (7.4)	4.7 (7.2)			
Max knee flexion	M	60.6 (4.7)	98.8 (8.0)	111.9 (7.4)	125.3 (7.0)	0.046	<0.001	0.019
	F	61.2 (4.8)	107.6 (4.0)	116.6 (4.7)	125.6 (7.0)			
Max ankle dorsiflexion	M	12.1 (3.8)	13.8 (4.1)	15.8 (3.7)	17.0 (4.7)	0.435	<0.001	0.141
	F	10.7 (3.7)	16.2 (4.6)	17.5 (4.7)	19.3 (5.5)			
Max ankle plantarflexion	M	15.4 (4.6)	12.6 (5.4)	13.2 (4.2)	12.3 (5.0)	0.084	< 0.001	0.006
	F	22.0 (4.9)	18.6 (8.5)	13.3 (5.5)	13.8 (4.7)			

M: male; F: female.

**Table 3:** Correlation between the joint angle and obstacle height.

	Male		Female	
	r	*p*	r	*p*
Max hip flexion angle	0.629	<0.001	0.632	<0.001
Max knee flexion angle	0.921	<0.001	0.932	<0.001
Max ankle dorsiflexion angle	0.436	<0.001	0.530	<0.001

**Table 4:** Mean and standard deviations of GRF values during the stance phase cycle. The last three columns show the *p*-values for the main effects of the gender and obstacle height and the gender × obstacle interaction, respectively.

GRF (% BW)		Unobstructed	Low	Medium	High	Gender	Obstacle	Gender × Obstacle
Fx_PP_	M	3.26 (1.81)	3.39 (1.67)	4.01 (1.88)	4.08 (1.82)	0.600	0.005	0.501
	F	2.81 (1.21)	3.49 (1.20)	3.53 (1.18)	3.73 (0.93)			
Fx_N1_	M	4.31 (0.95)	4.50 (1.19)	4.39 (1.26)	4.00 (1.21)	0.634	0.057	0.212
	F	4.50 (1.30)	3.94 (1.33)	4.04 (1.60)	3.79 (1.50)			
Fx_N2_	M	5.42 (1.13)	5.62 (1.37)	5.63 (1.73)	5.40 (1.34)	0.032	0.990	0.326
	F	4.50 (0.95)	4.31 (1.44)	4.30 (0.99)	4.63 (1.11)			
Fy_BF_	M	11.40 (2.92)	14.41 (2.79)	16.83 (3.16)	17.16 (3.04)	0.958	<0.001	0.094
	F	10.41 (4.05)	15.34 (4.31)	16.20 (4.30)	17.55 (4.49)			
Fy_PF_	M	18.59 (1.89)	22.90 (1.92)	22.70 (2.09)	22.19 (1.47)	0.257	<0.001	0.302
	F	19.80 (3.41)	24.86 (3.76)	23.21 (2.53)	22.84 (2.90)			
Fz_FP_	M	100.04 (7.78)	108.82 (8.44)	112.19 (8.37)	112.71 (7.67)	0.316	<0.001	0.577
	F	103.61 (8.26)	112.55 (7.33)	114.14 (7.76)	116.13 (9.26)			
Fz_SP_	M	108.79 (4.74)	115.66 (7.59)	115.97 (9.57)	116.48 (7.63)	0.043	<0.001	0.259
	F	115.43 (4.85)	123.05 (8.66)	121.94 (8.53)	120.96 (8.90)			
Fz_DF_	M	82.05 (5.39)	75.73 (4.68)	73.43 (6.06)	73.21 (5.74)	0.224	<0.001	0.632
	F	85.37 (6.63)	77.96 (6.66)	76.94 (8.14)	76.34 (8.51)			

Fx_PP_: initially occurring positive peak on the medial-lateral curve; Fx_N1_: first negative peak on the medial-lateral curve; Fx_N2_: second negative peak on the medial-lateral curve; Fy_BF_: initial braking force on the anterior–posterior curve; Fy_PF_: propulsion force on the anterior–posterior curve; Fz_FP_: first peak on the vertical curve; Fz_SP_: second peak on the vertical curve; Fz_DF_: downfall between the two peaks on the vertical curve; M: male; F: female.

## Data Availability

The original contributions presented in this study are included in the article. Further inquiries can be directed to the corresponding author.

## Abbreviations

The following abbreviations are used in this manuscript:

STT	Stance time of trialing leg
SWL	Swing time of leading leg
VCL	Vertical heel-clearance of the leading foot
VCT	Vertical heel-clearance of the trailing foot
HCL	Horizontal clearance of the leading foot
HCT	Horizontal clearance of the trailing foot
UM	Unobstructed gait for male participants
UF	Unobstructed gait for female participants
MM	Medium obstacle gait for male participants
MF	Medium obstacle gait for female participants
Fx_PP_	The initially occurring positive peak on the medial-lateral curve
Fx_N1_	The first negative peak on the medial-lateral curve
Fx_N2_	The second negative peak on the medial-lateral curve
Fy_BF_	The initial braking force on the anterior–posterior curve
Fy_PF_	The propulsion force on the anterior–posterior curve
Fz_FP_	The first peak on the vertical curve
Fz_SP_	The second peak on the vertical curve
Fz_DF_	The downfall between the two peaks on the vertical curve
